# sncRNA levels predict SNORD105B is a novel biomarker of chronic kidney disease risk and SGLT2 inhibitor response in type 2 diabetes

**DOI:** 10.1016/j.omtn.2026.102947

**Published:** 2026-05-01

**Authors:** Juliette A. de Klerk, Roderick C. Slieker, Joline W.J. Beulens, Janneke H.D. Peerlings, Hailiang Mei, Petra J.M. Elders, Anton Jan van Zonneveld, Daniël H. van Raalte, Roel Bijkerk, Leen M. ’t Hart

**Affiliations:** 1 Department of Cell and Chemical Biology, Leiden University Medical Center, 2333ZA Leiden, the Netherlands; 2 Department of Internal Medicine (Nephrology), Leiden University Medical Center, 2333ZA Leiden, the Netherlands; 3 Amsterdam Public Health Institute, Amsterdam UMC, 1081HV Amsterdam, the Netherlands; 4 Department of Epidemiology and Data Science, Amsterdam UMC, Location Vrije Universiteit, 1081HV Amsterdam, the Netherlands; 5 Julius Center for Health Sciences and Primary Care, University Medical Center Utrecht, 3508GA Utrecht, the Netherlands; 6 Department of Biomedical Data Sciences, Section Molecular Epidemiology, Leiden University Medical Center, 2333ZA Leiden, the Netherlands; 7 Department of General Practice and Elderly Care Medicine, Amsterdam Public Health Research Institute, Amsterdam UMC, Location VUmc, 1081HV Amsterdam, the Netherlands; 8 Diabetes Center, Department of Internal Medicine, Amsterdam University Medical Centers, 1081HV Amsterdam, the Netherlands

**Keywords:** MT: Non-coding RNAs, small non-coding RNAs, small nucleolar RNAs, chronic kidney disease, type 2 diabetes, sodium-glucose transport 2, SGLT2, inhibitors

## Abstract

Chronic kidney disease (CKD) is a common complication of type 2 diabetes, characterized by reduced kidney function and/or albuminuria, yet its progression varies widely among individuals. While sodium-glucose cotransporter-2 (SGLT2) inhibitors are known to protect against kidney decline, the molecular mechanisms underlying their renoprotective effects remain incompletely understood. Circulating small non-coding RNAs (sncRNAs), particularly microRNAs, have been linked to CKD but the contribution of other sncRNA classes is less explored. We profiled plasma sncRNAs in 263 participants with type 2 diabetes from the Hoorn DCS cohort without CKD at baseline, followed for ∼9 years (*n*_control_ = 122, *n*_case_ = 141). sncRNA profiling was also performed before and after treatment of SGLT2 inhibitors in three trials (*n* = 65, total) to assess drug-induced molecular changes in the circulation. Eleven sncRNAs were nominally associated with incident CKD, most strongly *SNORD12C* and *SNORD105B*. In an independent SGLT2 inhibitor trial, exploratory analyses identified 34 sncRNAs that changed following treatment, including *SNORD105B*. Preliminary analyses linked these snoRNAs to co-regulated proteins, suggesting potential functional relevance. Our results identify snoRNAs, particularly *SNORD105B*, as potential novel markers of CKD risk and SGLT2 inhibitor response in type 2 diabetes, revealing an unexplored sncRNA axis and providing a foundation for future functional studies into their mechanistic role in CKD progression.

## Graphical abstract

**Figure:**
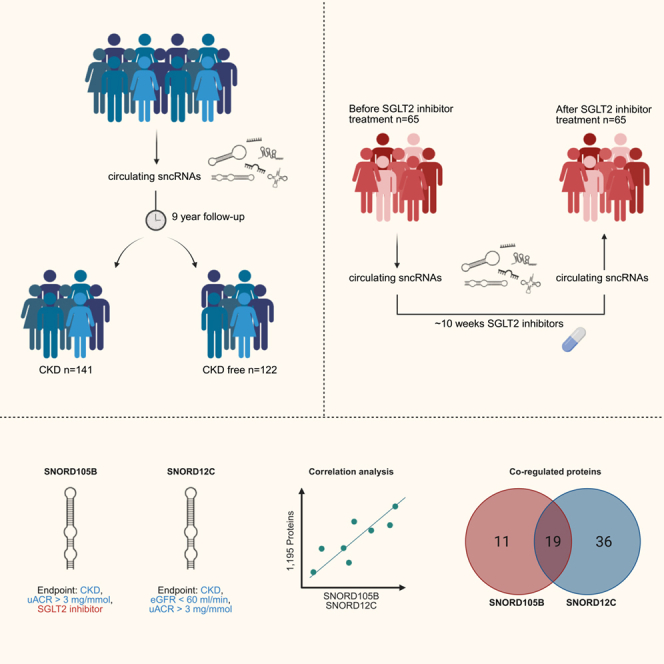

## Teaser

Chronic kidney disease risk and response to SGLT2 inhibitors in type 2 diabetes are associated with circulating small non-coding RNAs, particularly *SNORD12C* and *SNORD105B*. These findings uncover a previously unrecognized small non-coding RNA axis and provide new insight into mechanisms underlying kidney disease progression and renoprotective therapy.

## Introduction

Chronic kidney disease (CKD) is a heterogeneous disorder affecting kidney structure and function. It is one of the most common complications in type 2 diabetes, with a prevalence of around 27%.^1^ CKD is characterized by reduced estimated glomerular filtration rate (<60 mL/min/1.73 m^2^ eGFR) and albuminuria (urine albumin-creatinine ratio, uACR >3 mg/mmol). Nonetheless, while some individuals develop complications like CKD early in the disease trajectory, others will never develop this complication. The mechanisms underlying CKD development in type 2 diabetes are not fully understood.^2^

Small non-coding RNAs (sncRNAs), including microRNAs (miRNAs), PIWI-interacting (piRNAs), and small nucleolar RNAs (snoRNAs), represent a unique and novel source to provide new biological insight in the mechanisms of CKD in diabetes. Previous studies have shown that miRNAs transported in vesicles through the bloodstream can influence the fate and function of recipient cells and tissues, including kidney cells.^3^^,^^4^ This supports the concept that sncRNAs, like miRNAs, serve as mediators of inter-organ communication and play critical roles in maintaining kidney function. However, very little is known about the role of other sncRNA types in multi-system communication. We recently found evidence that circulating sncRNAs, belonging to various sncRNA classes, are associated with kidney function (eGFR, uACR) and prevalent diabetic CKD.^5^ Interestingly, these sncRNAs are often expressed in tissues other than the kidney suggestive of a role in multi-system communication in kidney dysfunction.

Sodium-glucose cotransporter-2 (SGLT2) inhibitors are drugs used to reduce hyperglycemia in type 2 diabetes patients by increasing urinary glucose excretion via the inhibition of glucose reabsorption in the kidney proximal tubules.^6^ While SGLT2 inhibitors are mainly used in people with type 2 diabetes, they have also proven effective in slowing CKD progression in patients with and without diabetes.^7^ Mechanisms underlying the beneficial effects of SGLT2 inhibitors on the kidney have not been fully elucidated.^8^ Due to the decreased reabsorption of glucose, tubular glomerular feedback may be restored. This leads to a reduction in intra-glomerular pressure and a decrease in glomerular hyperfiltration. However, these mechanisms are unlikely to fully explain the observed kidney benefits.

In the current study we measured the circulating sncRNA transcriptome in participants from the prospective Hoorn Diabetes Care System (Hoorn DCS) study to elucidate if circulating sncRNAs are associated with incident CKD in people with type 2 diabetes during follow-up. In addition, we used existing SGLT2 inhibitor trial data to study the effect of SGLT2-i treatment on sncRNA levels.

## Results

### Baseline characteristics of incident CKD study

The median (IQR) age of the individuals was 64.5 (59.3–69.5) years (Table S1). In total, 43.0% of the population was female. The population was on average overweight (BMI = 29.3 [26.8–32.6]) with well-controlled HbA_1c_ levels, 48.6 (43.2–56.3), 6.6% (6.1%–7.3%). Although in the normal range, the controls and cases had a slightly different baseline eGFR (cases = 75.2 [68.1–86.4], controls = 88.6 [80.8–95.4]) and uACR (cases = 0.7 (0.4–1.2), controls = 0.4 (0.0–0.6), Figure S1).

### Different classes of sncRNAs are associated with incident CKD

**Figure 1:**
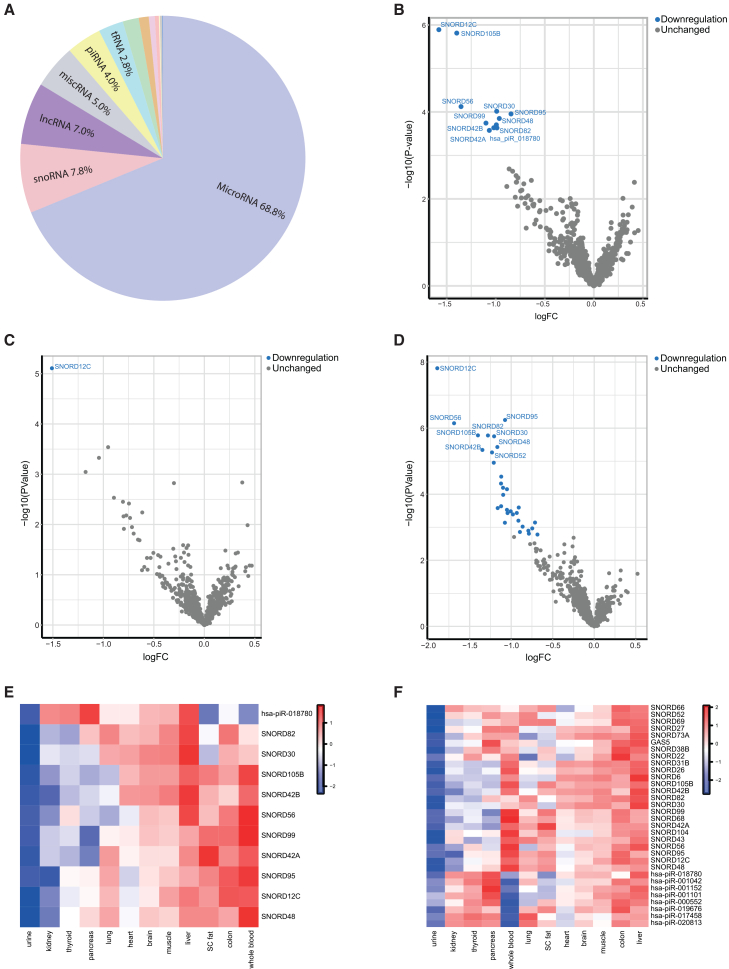
Circulating sncRNAs associated with incident CKD and its clinical endpoints (A) The distribution of sncRNA biotypes measured by bulk RNA-sequencing in the incident CKD study. (B) A volcano plot displays sncRNAs associated with incident CKD in the base model (FDR < 0.05). (C) A volcano plot displays sncRNAs associated with reduced kidney function (base model), defined as an eGFR < 60 ml/min/1.73 m^2^. (D) A volcano plot displays sncRNAs associated with elevated albuminuria (uACR > 3 mg/mmol) in the base model. (E) Tissue-specific expression patterns of the CKD-associated sncRNAs. Data was *z* scaled. (F) Tissue-specific expression patterns of sncRNAs associated with the uACR endpoint. Data was *z* scaled.

To investigate circulating sncRNAs that associate with future CKD in this cohort, 881 sncRNAs were included in the differentially expression analysis based on sufficient expression levels. For the larger part, these included miRNAs, followed by snoRNAs, long non-coding RNAs (lncRNAs), miscellaneous RNA (miscRNAs), piRNAs and the remaining sncRNAs (Figure 1A; Table S2). In total, 11 sncRNAs were significantly associated with incident CKD in the base model, including adjustment for baseline eGFR. These included 10 snoRNAs as follows: *SNORD105B*, *SNORD12C*, *SNORD95*, *SNORD30*, *SNORD56*, *SNORD99*, *SNORD42B*, *SNORD48*, *SNORD82* and *SNORD42A*, and 1 piRNA; *hsa-piR-018780* (Figure 1B; Table S3). There is a high degree of correlation among most of the sncRNAs associated with CKD (Figure S2). None of the sncRNAs remained significant in the fully adjusted model, including adjustment for baseline systolic blood pressure (SBP), eGFR, uACR, and HbA_1c_ (Table S3). Furthermore, one sncRNA, *SNORD12C*, was associated with an eGFR <60 mL/min/1.73 m^2^, but this association was not significant in the fully adjusted model (Figure 1C; Table S4). Thirty-one sncRNAs are significantly associated with albuminuria (uACR >3 mg/mmol) (Figure 1D; Table S5). These included 22 snoRNAs (including *SNORD105B* and *SNORD12C*), 8 piRNAs, and 1 lncRNA fragment (*GAS5*). Of which, six snoRNAs remained significant in the fully adjusted model: *SNORD105B*, *SNORD95*, *SNORD42B*, *SNORD48*, *SNORD82*, and *SNORD52* (Table S5). *SNORD12C* was significantly associated with all three endpoints in the base model: incident CKD, eGFR <60 mL/min/1.73 m^2^ and albuminuria (uACR >3 mg/mmol). Interestingly, lncRNA *GAS5* is a host gene for 10 snoRNAs. The reads mapped to GAS5 mainly map to three of the ten snoRNAs: *SNORD81* (chr1:173864146–173864222), *SNORD44* (chr1:173865968–173866028) and *SNORD74* (chr1:173867674–173867745) (Figure S3). Since sncRNAs may be involved in cross-organ cell-cell communication, and to determine potential tissue origin, we next analyzed tissue expression of the CKD-associated sncRNAs. Most of them are expressed in tissues other than the kidney, including whole blood, colon, subcutaneous (s.c.) fat, and the liver (Figure 1E). The 32 sncRNAs associated with albuminuria are mainly expressed in the liver and colon. In addition, the piRNAs are also enriched in the pancreas and the snoRNAs in whole blood (Figure 1F).

### Baseline characteristics of SGLT2 inhibitor study

Next, we aimed to assess the impact of SGLT2 inhibition on the circulating sncRNA transcriptome in patients with type 2 diabetes with preserved kidney function. Characteristics of the participants of the SGLT2 inhibition treatment study are given in Table S6. The mean (SD) age of the individuals was 64.6 ± 6.5 years. In total 18.5% of the population was female. Participants were obese (BMI 31.0 ± 3.9) with diabetes (HbA_1c_ 55.0 ± 26.6 mmol/mol) and an average disease duration of 10.3 ± 5.5 years. Participants showed no evidence of kidney disease, with a mean measured glomerular filtration rate (mGFR) of 112.3 ± 26.1.

### Different classes of sncRNAs are associated with SGLT2 inhibition treatment

**Figure 2:**
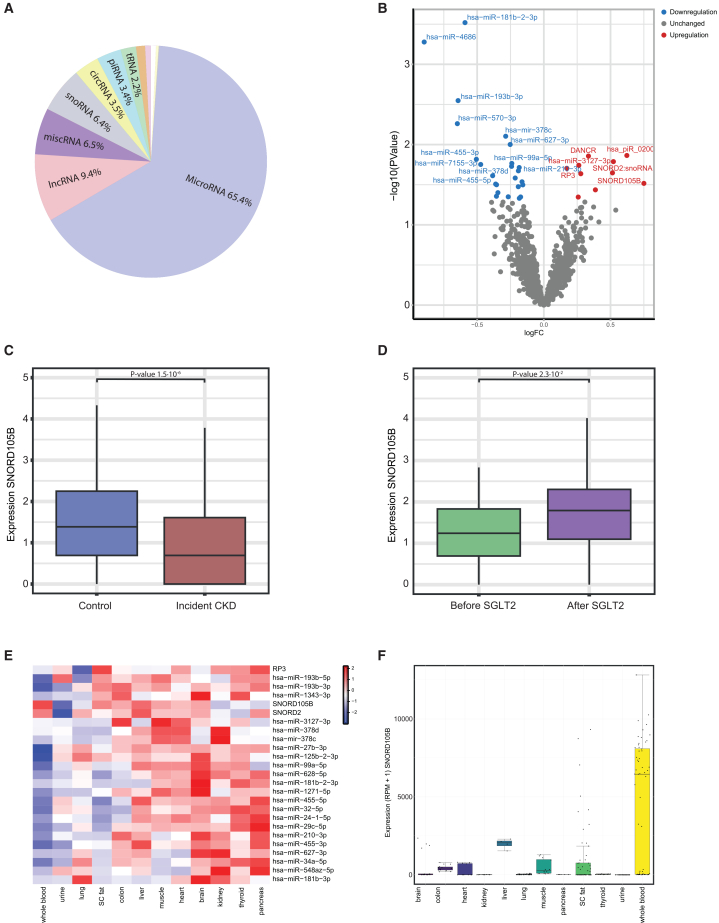
Effects of SGLT2 inhibitor treatment on circulating sncRNAs and *SNORD105B* expression (A) The distribution of sncRNA biotypes measured by bulk RNA-sequencing in the SGLT2 inhibitor treatment study. (B) A volcano plot presents sncRNAs significantly (nominal) altered following SGLT2 inhibitor treatment comparing before and after SGLT2 inhibitor treatment (*p* value < 0.05). (C) *SNORD105B* expression is compared between individuals with incident CKD and matched controls in the incident CKD study (*p* value < 0.05). (D) *SNORD105B* expression levels before and after SGLT2 inhibitor treatment (*p* value < 0.05). (E) Tissue-specific expression patterns of these SGLT2-associated sncRNAs. Data was *z* scaled. (F) Tissue expression of *SNORD105B*. Data was *z* scaled.

For the SGLT2 inhibitor treatment study, 1,113 sncRNAs were included in the differentially expression analysis. These were similar in types of sncRNAs and distribution as for the incident CKD study (Figure 2A; Table S7). The expression of 34 sncRNAs change upon SGLT2 inhibition treatment. These included 26 miRNAs (a.o. *hsa-miR-181b-2-3p*, *hsa-miR-4686*, *hsa-miR-193b-3p*), 3 snoRNAs (*SNORD2*, *SNORD105B*, *SNORD57*), 2 lncRNA (*DANCR*, *RP3*), 2 circRNAs (*hsa_circ_000745*, *hsa_circ_001799*), and 1 piRNA (*hsa_piR_020008*) (Figure 2B; Table S8). Sensitivity analyses were performed by stratifying the SGLT2 inhibitor cohort according to treatment regimen (metformin + dapagliflozin, metformin + empagliflozin, and metformin + linagliptin + empagliflozin) for those 34 nominal significant sncRNAs. Effect sizes were generally similar across groups, although most associations did not reach statistical significance (Tables S9–S11; Figure S4). These findings should therefore be interpreted with caution, as the lack of significance is likely driven by the very small sample sizes within each treatment group. Although no associations remained significant after correction for multiple testing, *SNORD105B* emerged as a sncRNA of particular interest. This snoRNA was downregulated in individuals with incident CKD (base model), uACR >3 mg/mmol (base and fully adjusted model) and was upregulated following SGLT2 inhibitor treatment (nominal) (Figures 2C and 2D). Most of the SGLT2 inhibitor-associated sncRNAs are expressed in multiple tissues, including the brain, heart, muscle, liver, pancreas, and kidney (Figure 2E). *SNORD105B* is abundantly expressed in whole blood, besides expression in liver, s.c. fat, colon, muscle, brain and heart (Figure 2F).

### Co-regulated proteins

Finally, because the roles of most snoRNAs identified in our study remain unclear, we explored their potential targets or co-regulated proteins and pathways by correlating their expression levels with those of 1,195 plasma proteins. For *SNORD105B*, we identified 30-associated proteins, with the strongest correlation observed for CCL27 (beta = 0.07, P_FDR_ = 7.5 × 10^−5^) (Table S12; Figure S5). Subsequent pathway enrichment analysis of these proteins highlighted a significant overrepresentation in the extracellular region (false discovery rate, FDR = 5.1 × 10^−11^) and in processes related to cell signaling, particularly receptor-ligand interactions (FDR = 1.3 × 10^−5^) (Figure S6). Applying the same approach to *SNORD12C* revealed 55-associated proteins, with the strongest association found for CCL28 (beta = 0.20, P_FDR_ = 7.4 × 10^−3^) (Table S12; Figure S7). Enrichment analysis of these proteins again demonstrated a pronounced presence in the extracellular region (FDR = 2.4 × 10^−11^) and in binding-related functions, particularly protein binding (FDR = 3.7 × 10^−10^) (Figure S8). Notably, 19 proteins overlapping between *SNORD105B* and *SNORD12C* suggest that these snoRNAs may share common regulatory targets or participate in related pathways (Figure S9; Table S12).

## Discussion

In this study, we used two independent studies to investigate the role of circulating sncRNAs in the development of CKD in type 2 diabetes and the response to SGLT2 inhibitor treatment in type 2 diabetes patients with preserved kidney function. Notably, the prospective cohort included a 9 year follow-up period, providing an opportunity to explore long-term associations between baseline sncRNA levels and future kidney outcomes in this population. We identified 11 sncRNAs associated with incident CKD, one sncRNA associated with reduced kidney function (eGFR <60 mL/min/1.73 m^2^), and 31 sncRNAs associated with albuminuria (uACR >3 mg/mmol) in the base model. After full adjustment for baseline SBP, eGFR, uACR, and HbA1c, six snoRNAs remained significantly associated with albuminuria: *SNORD105B*, *SNORD95*, *SNORD42B*, *SNORD48*, *SNORD82*, and *SNORD52*. Following SGLT2 inhibitor treatment, expression levels of 34 sncRNAs changed; however, none remained statistically significant after correction for multiple testing likely due to low a priori power. Furthermore, we observed that these sncRNAs are expressed across multiple tissues, suggesting a potential role in inter-organ communication. Notably, *SNORD105B* was downregulated in participants with type 2 diabetes, who later developed CKD (base model) and associated with albuminuria in both the base and fully adjusted models, while it was upregulated following SGLT2 inhibitor treatment (nominal), making it the central focus of this study. While most previous research has concentrated on miRNAs, our findings highlight that snoRNAs, rather than miRNAs, showed the strongest associations with a number of kidney outcomes.^9^ This is also in line with our previous study investigating sncRNAs in prevalent CKD in people with type 2 diabetes.^5^ This suggests a potentially underexplored role for snoRNAs in the pathophysiology of CKD and its modulation by SGLT2 inhibition.

snoRNAs are highly abundant in the nucleoli of eukaryotic cells, where they play a key role in the chemical modification of ribosomal RNA (rRNA). Traditionally, snoRNAs are best known for guiding site-specific modifications on rRNA, including 2′-O-methylation and pseudouridylation, processes that help stabilize rRNA structure and protect it from degradation by nucleases.^10^ The focus of this study, *SNORD105B*, belongs to the C/D box snoRNA family. More recently, snoRNAs have also been implicated in additional RNA processing events, such as the modification of transfer RNA (tRNA and messenger RNA (mRNA), regulation of alternative splicing, and even exhibiting miRNA-like regulatory functions, a process which is mostly observed for C/D box snoRNAs.^11^^,^^12^^,^^13^ Genomically, most snoRNAs are encoded within the intronic regions of protein-coding or non-coding host genes. A well-known example is the long non-coding RNA *GAS5*, which serves as a host gene for 10 different snoRNAs.^14^

Building on these expanding non-canonical roles of snoRNAs, we next explored whether *SNORD105B* and *SNORD12C* are associated with specific protein networks as a first attempt to give directions for future functional studies. By correlating snoRNA expression levels with circulating plasma proteins, we identified distinct sets of proteins, whose abundance covaried with *SNORD105B* and *SNORD12C*. For *SNORD105B*, correlated proteins were strongly enriched for extracellular localization and for molecular functions related to cell signaling, particularly receptor-ligand interactions and growth factor activity. This pattern suggests that *SNORD105B* expression may reflect, or potentially contribute to, biological processes involved in intercellular communication rather than exclusively intracellular RNA modification. The strong association with chemokines such as CCL27 and CCL28 further supports a link to immune-related signaling pathways. CCL27 may play a role in kidney disease by promoting immune cell recruitment. Genetic evidence indicates that systemic inflammatory regulators, including chemokines like CCL27, can causally influence CKD risk.^15^ Elevated CCL27 levels are also associated with acute kidney injury after surgery.^16^ A similar enrichment for extracellular proteins was observed for *SNORD12C*, with binding-related molecular functions, particularly protein binding, emerging as the most prominent category. Notably, a substantial fraction of correlated proteins overlapped between *SNORD105B* and *SNORD12C*, suggesting that these snoRNAs may participate in shared regulatory programs or respond to common upstream stimuli. While these correlations do not imply direct molecular interactions, they are consistent with an emerging view in which snoRNAs, especially C/D box snoRNAs, are integrated into broader regulatory networks that extend beyond the nucleolus and may influence or mirror extracellular signaling states.

Limitations of this study include the relatively modest changes observed in the expression of the investigated sncRNAs. However, such subtle differences may be expected, given the regulatory nature of sncRNAs, which often exert their effects through fine-tuning gene expression and functioning in concert within broader regulatory networks.^17^^,^^18^ A further limitation relates to differences in baseline characteristics between cases and controls in the case-control study. Although all participants were free of CKD at baseline, cases already showed lower eGFR and higher uACR compared with controls, suggesting that underlying differences in kidney health may have been present prior to outcome ascertainment. Consequently, some of the observed associations may reflect early or subclinical disease processes rather than purely predictive biological signals. Moreover, after full adjustment for baseline eGFR, uACR, SBP, and HbA1c, nearly all associations were attenuated and no longer statistically significant, further indicating that baseline differences in clinical risk factors may explain part of the observed effects. For the SGLT2 treatment analysis, most of the 34 nominally significant sncRNA associations did not remain statistically significant in the per-treatment regimen analysis, likely due to the small sample sizes within each subgroup, despite generally similar effect sizes across regimens. Therefore, reduced statistical power should be considered when interpreting the findings. Finally, the co-regulated proteins were identified using samples from the same cohort (Hoorn DCS), consisting exclusively of individuals with type 2 diabetes but derived from participants with prevalent CKD rather than incident CKD. Although this limits direct comparability, these analyses remain informative and provide insight into potential biological functions and pathways associated with the identified snoRNAs.

### Conclusion

Our findings highlight *SNORD105B*, a circulating sncRNA, associated with incident CKD in the base model and with albuminuria in both the base and fully adjusted models, as well as with response to SGLT2 inhibition (nominal). Our findings provide a rationale for future functional investigations to determine whether *SNORD105B* has a function in development of CKD and could be targeted or leveraged in therapeutic strategies for CKD.

## Materials and methods

### Subjects

The DCS cohort is an open prospective study that was initiated in 1998 and includes individuals with type 2 diabetes living in the northwest region of the Netherlands. Participants attended annual check-ups at the DCS (1998–2019), during which their diabetes status is routinely monitored. These visits include repeated assessments of anthropometric measures and laboratory parameters. In addition, participants were invited to join the Hoorn DCS biobank. After obtaining informed consent, serum and plasma samples were collected and stored for future research purposes. Biobanking of blood samples was carried out in two phases, first in 2008–2009 and later in 2012–2014. CKD risk stage is calculated using the Kidney Disease Improving Global Outcomes (KDIGO) criteria based on the combination of eGFR and uACR (0 = low risk; 3 = very high risk).^19^ For the current study, we used a matched case-control design nested in the whole DCS biobank (*n* = 263) using the following inclusion criteria: CKD stage 0 at the time of their first biobank sample and at least the year before. Cases were those who developed CKD during follow-up (CGA risk stage ≥2 (high risk) in at least two consecutive years, *n* = 141), whereas controls were sex- and diabetes-duration-matched persons, who remained stable in stage 0 during at least three yearly follow-up visits (*n* = 122). All laboratory measurements were done on samples taken in fasted state. Details of the laboratory measurements have been described in van der Heijden et al.^20^ The study has been approved by the Ethical Review Committee of the VU University Medical Center, Amsterdam.

### SGLT2 inhibitors

For the analysis of therapeutic interventions with SGLT2 inhibitors and the sncRNA transcriptome, we measured sncRNA transcriptomes in people with type 2 diabetes from three clinical trials before and after treatment with an SGLT2 inhibitor with similar inclusion criteria. In these trials, people were treated for ∼10 weeks with either metformin and dapagliflozin (RED, *n* = 24),^21^ metformin and empagliflozin (RACE_1, *n* = 20), or metformin, linagliptin, and empagliflozin (RACE_2, *n* = 21).^22^ The three studies complied with the Declaration of Helsinki and Good Clinical Practice guidelines and were registered at ClinicalTrials.gov (NCT02682563 and NCT03433248). Participants were deeply phenotyped with respect to mGFR, kidney hemodynamic function, blood pressure, sodium excretion, and cardiovascular function. In total, we included 65 participants with type 2 diabetes from whom two samples are available for sequencing (pre- and on-treatment).

### RNA isolation and sequencing

RNA was extracted from 400 to 800 μL of citrate plasma using a commercially available kit for cell-free RNA isolation (Quick-cfRNA Serum and Plasma Kit, Zymo Research, Irvine, CA, USA). Following quality assessment, a total of 263 samples from the incident CKD cohort and 130 samples from the SGLT2 cohort were selected for sncRNA sequencing (sncRNA-seq). Library preps were constructed using the NEBNext Multiplex Small RNA Library Prep Set for Illumina (New England Biolabs, Ipswich, MA, USA). To enrich for sncRNAs (∼20–100 nucleotides), fragment size selection between 120 and 200 nucleotides was carried out using a Pippin Prep system (Sage Science, Beverly, MA, USA), as described earlier.^23^ Sequencing was performed on the Illumina NovaSeq 6000 platform using v.2.5 reagent kits with 150 cycle paired-end reads (PE150), generating ∼3 Gb of data per sample (approximately 10 million paired-end reads). The exceRpt pipeline developed by the NIH Extracellular RNA Communication Consortium (ERCC)^24^ was used for quality control, to process the data and generate RNA abundance estimates for miRNA, piRNA, lncRNA, tRNA fragments, Y_RNA (fragments), snRNA, snoRNA, scaRNA, and various other sncRNA species using miRBase v.22,^25^ piRNABank v.1,^26^ Gencode v.38,^27^ circBase.^28^ and GtRNAdb.^29^ Two samples of the SGLT2 study did not pass the quality control (QC) threshold and were excluded.

### Tissue expression of sncRNAs

Raw sncRNA-seq datasets from a range of metabolic tissues were obtained from the publicly accessible Gene Expression Omnibus (GEO).^30^ The analyzed tissues included kidney, thyroid, pancreas, colon, liver, heart, muscle, brain, s.c. white adipose tissue, as well as whole blood, and urine (Table S13). Processing of the sequencing data was performed using the exceRpt pipeline, as described earlier. These datasets were applied to assess the relative abundance of sncRNAs across tissues. For visualization, values were *z* scaled in the figures.

### Plasma proteomics

Plasma proteomic data were available for 589 participants from the Hoorn DCS study and were generated using the SomaLogic SOMAscan platform (Boulder, CO, USA), as previously described.^31^ After quality control, measurements for 1,195 proteins were retained for analysis. For a subset of 199 participants, matched small non-coding RNA sequencing (sncRNA-seq) data from the same sampling date were also available.^5^ Notably, no individuals with incident CKD were present in this subset nor was there any overlap with the current study. Associations between the sncRNA transcriptome and the plasma proteome were assessed using linear regression models, with log-transformed protein levels as the outcome and sncRNA expression levels as predictors, adjusting for age, BMI, sex, and technical covariates. Multiple testing was addressed using the Benjamini-Hochberg procedure, and a FDR < 0.05 was considered statistically significant. Pathway enrichment analysis was performed using STRING (v.12.0), focusing on Gene Ontology Cellular Component and Molecular Function terms.

### Statistical analysis

Differences between baseline characteristics of cases and controls for the CKD study were determined with the Mann-Whitney *U* test and categorical values with the chi-squared test. A *p* value below 0.05 was considered significant. Differential expression analysis of the CKD study was performed using a quasi-likelihood (QL) F-test with R-package *edgeR* (v.4.7.2). Lowly expressed sncRNAs were filtered out (threshold of mean ≥ 5 copies). The base model was adjusted for baseline eGFR. A fully adjusted model was also adjusted for baseline SBP, uACR, and HbA_1c_. Age and sex were not included in the models because they were already included in the eGFR (CDK-EPI) formula and patients were sex matched. We tested for the primary outcome CKD (stage ≥2) and multiple secondary outcomes: eGFR <60 mL/min/1.73 m^2^ and uACR >3 mg/mmol. Similar to the CKD study, for the SGLT2 study a QL F-test was used to find differentially expressed sncRNAs after treatment with SGLT2 inhibitors. The three studies were pooled and the model was adjusted for age, sex, and treatment regimen (metformin + dapagliflozin, metformin + empagliflozin, or metformin + linagliptin + empagliflozin). In addition, sensitivity analyses were conducted on the 34 nominally significant sncRNAs, in which each treatment regimen was analyzed separately to assess whether associations differed between regimens. SncRNAs were considered differentially expressed if an observed difference between two conditions was statistically significant based on an FDR-adjusted *p* value below 0.05. All analyses were performed using R statistics (v.4.3.2). Figures were produced using the R package *NMF* (v.0.26.0) and *ggplot2* (v.3.4.4).

## Data availability

The datasets generated and/or analyzed during the current study are not publicly available due to restrictions in the informed consent but are available from the corresponding author on reasonable request and after signing an appropriate collaboration and data transfer agreement.

## Acknowledgments

We would like to thank all staff and participants of the Hoorn Diabetes Care System for their support and participation. This work was supported by a diabetes breakthrough grant from the Dutch Diabetes Research Foundation and ZonMw (grant number 459001015) and a grant from the EFSD/Boehringer Ingelheim European Research Programme on “Multi-System Challenges in Diabetes” 2021.

## Author contributions

J.A.d.K., R.C.S., D.H.v.R., and L.M’t.H. designed the study, performed the analyses, and drafted the manuscript; L.M’t.H. and D.H.v.R. contributed to the data acquisition and project logistics; J.HD.P. provided technical assistance; P.JM.E. and J.WJ.B. contributed to data acquisition; H.M. was involved in the preprocessing of the RNA-seq data; A.J.v.Z. and R.B. contributed to the data interpretation. All authors critically revised the manuscript and approved the final version. L.M’t.H. is the guarantor of the work.

## Declaration of interests

The authors declare no competing interests.

## Declaration of generative AI and AI-assisted technologies in the writing process

During the preparation of this work, the authors used ChatGPT in order to improve writing. After using this tool/service, the authors reviewed and edited the content as needed and take full responsibility for the content of the publication.

## Supplemental information

Data S1. Tables S3–S6 and S8–S12

Document S2. Article plus supplemental information
