# Supplementary material for: sncRNA levels predict SNORD105B is a novel biomarker of chronic kidney disease risk and SGLT2 inhibitor response in type 2 diabetes
Source: Mol Ther Nucleic Acids. 2026 May 1;37(2):102947. doi: 10.1016/j.omtn.2026.102947 (PMC13223920; doi:10.1016/j.omtn.2026.102947)
Supplement: Document S1. Figures S1–S9 and Tables S1, S2, S7, and S13 [file mmc1.pdf]

OMTN, Volume *37*

Supplemental information

sncRNA levels predict SNORD105B is a novel biomarker of chronic kidney disease risk and SGLT2 inhibitor response in type 2 diabetes

Juliette A. de Klerk, Roderick C. Slieker, Joline W.J. Beulens, Janneke H.D. Peerlings, Hailiang Mei, Petra J.M. Elders, Anton Jan van Zonneveld, Daniël H. van Raalte, Roel Bijkerk, and Leen M. 't Hart

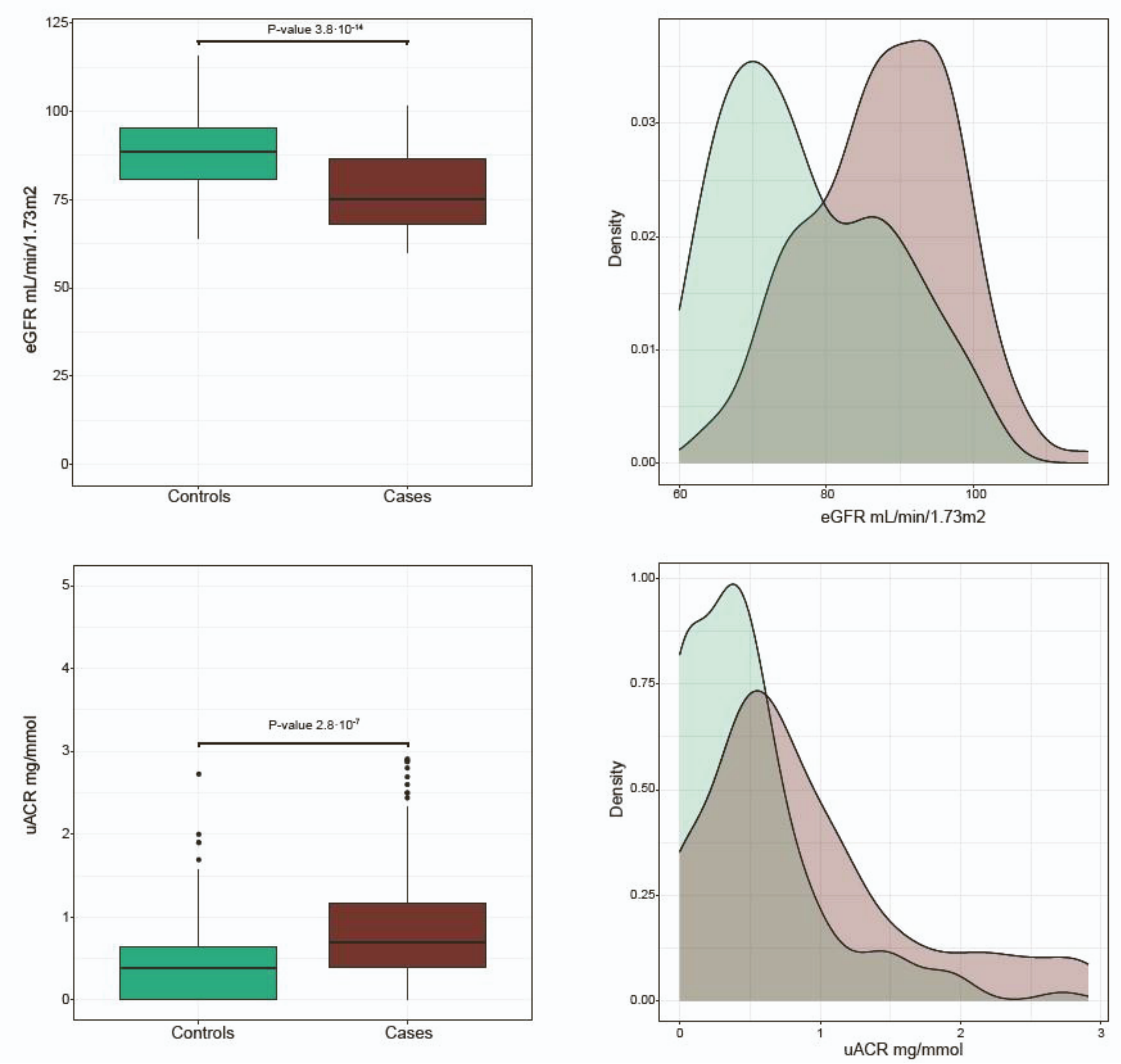

Figure S1. Comparison of baseline kidney function (eGFR) and kidney damage marker (uACR) between cases and controls. Both boxplot and density plot of baseline eGFR in controls and cases. Cases show significantly lower eGFR compared to controls (P-value = $3.8 \cdot 10^{-4}$). Both boxplot and density plot of baseline uACR in controls and cases. Cases show significantly higher uACR compared to controls (P-value = $2.8 \cdot 10^{-7}$).

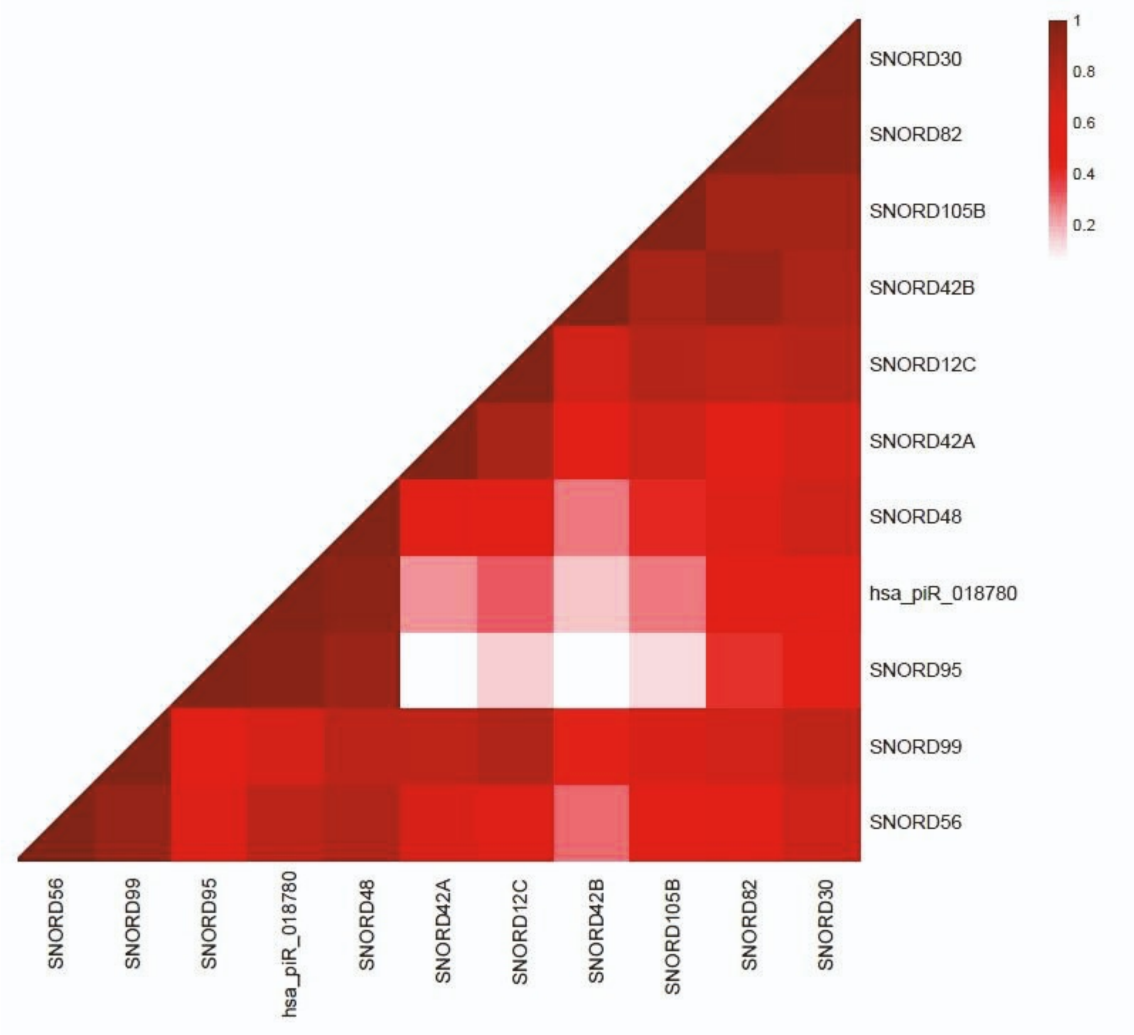

Figure S2. Correlation matrix of CKD associated sncRNAs. Pairwise correlations between the indicated snoRNAs and piRNA (hsa_piR_018780) are shown as a heatmap. The color scale represents the strength of the correlation, ranging from strong negative correlations (blue) to strong positive correlations (red).

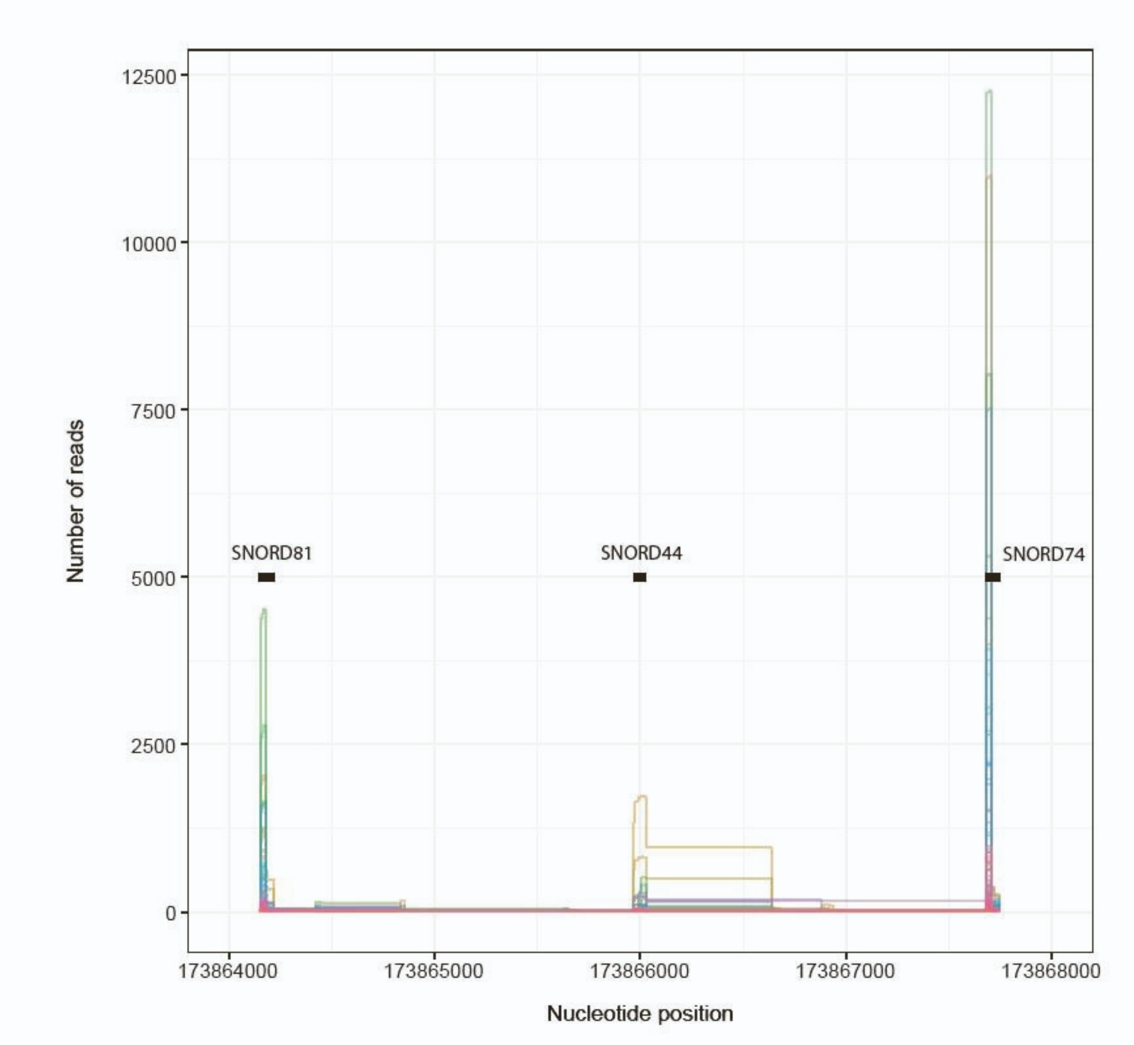

Figure S3. Read coverage across the *GAS5* locus. Coverage plot showing the number of sequencing reads mapped to nucleotide positions across the *GAS5* gene. The x-axis represents the genomic coordinates (nucleotide position), and the y-axis represents the number of aligned reads. Different coloured lines indicate coverage profiles from individual samples. Peaks correspond to regions of high read density, suggesting differential transcriptional activity across *GAS5*. Black bars represent the location of three snoRNAs: *SNORD81* (chr1:173864146-173864222), *SNORD44* (chr1:173865968173866028) and *SNORD74* (chr1:173867674-173867745).

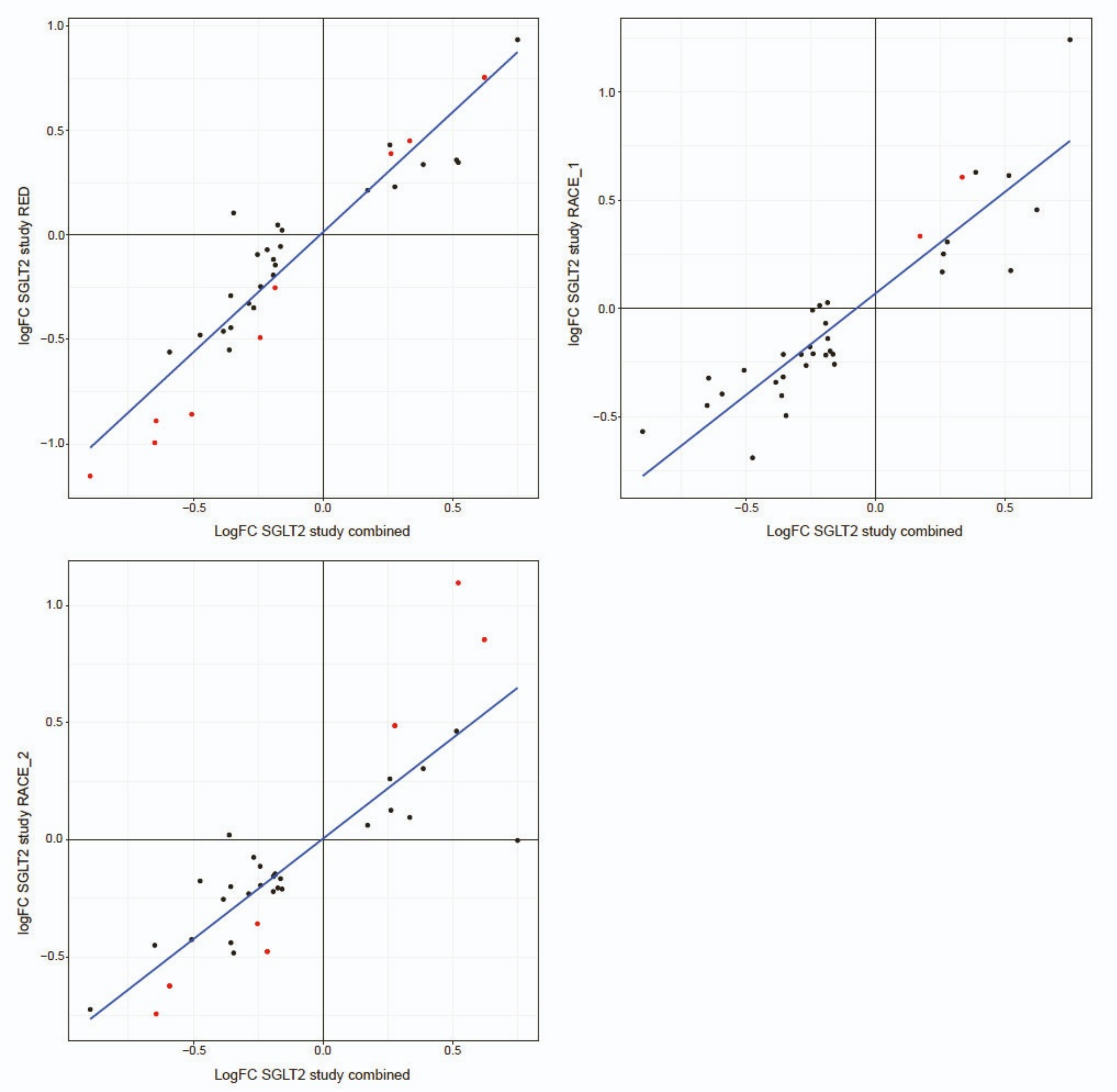

Figure S4. Beta–beta plot of 34 sncRNAs identified in the SGLT2 inhibitor treatment analysis, compared across three treatment regimens. In these trials, participants were treated for approximately 10 weeks with metformin plus dapagliflozin (RED, n = 24), metformin plus empagliflozin (RACE-1, n = 20), or metformin, linagliptin, and empagliflozin (RACE-2, n = 21). The plot displays the effect sizes (logFC) of the 34 nominally significant sncRNAs from the combined analysis against those observed in the SGLT2 inhibitor analysis, stratified by treatment regimen. Red dots represent nominal significant sncRNA in the treatment regimen analysis.

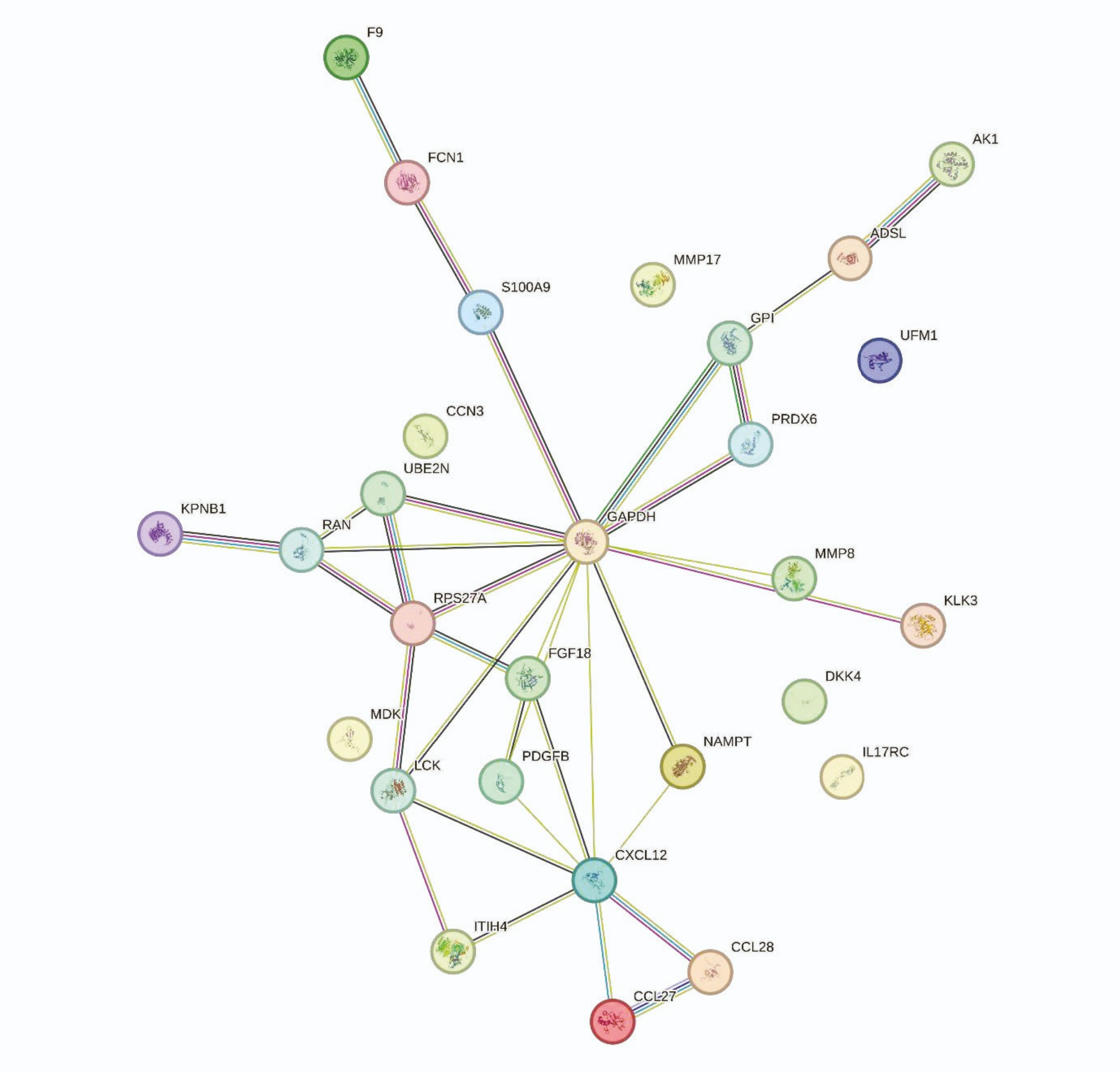

Figure S5. STRING protein-protein network of co-regulated proteins associated with *SNORD105B*. STRING protein–protein interaction network constructed from proteins significantly correlated with *SNORD105B* expression in plasma (n = 1,195 proteins screened; 30 significant associations).

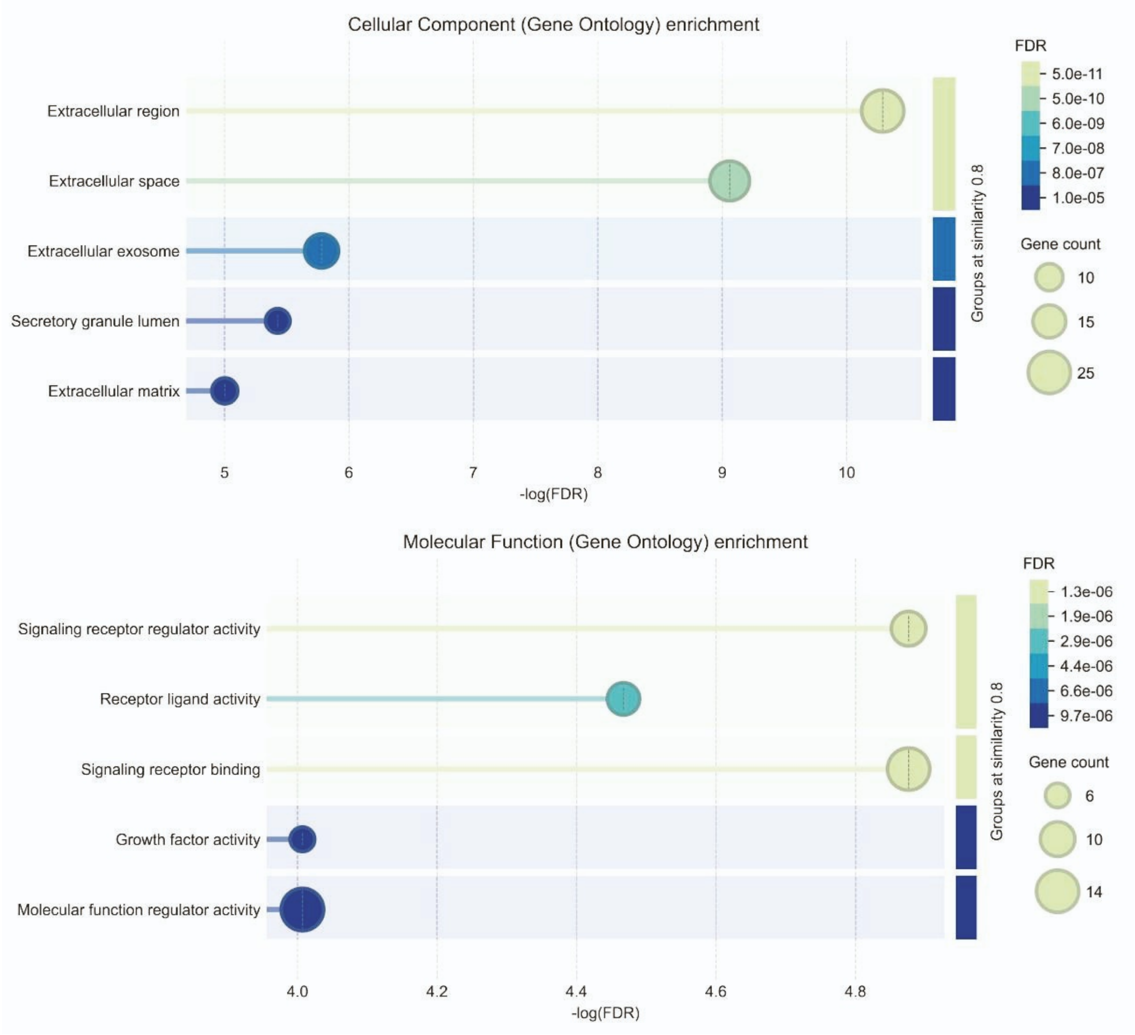

Figure S6. Enriched pathways associated with co-regulated proteins of *SNORD105B*. Correlation analysis between *SNORD105B* expression and 1,195 plasma proteins identified 30 proteins co-regulated with *SNORD105B*. Pathway enrichment analysis of *SNORD105B*-associated proteins revealed significant enrichment for extracellular region localization and signalling-related functions, particularly receptor–ligand interactions.

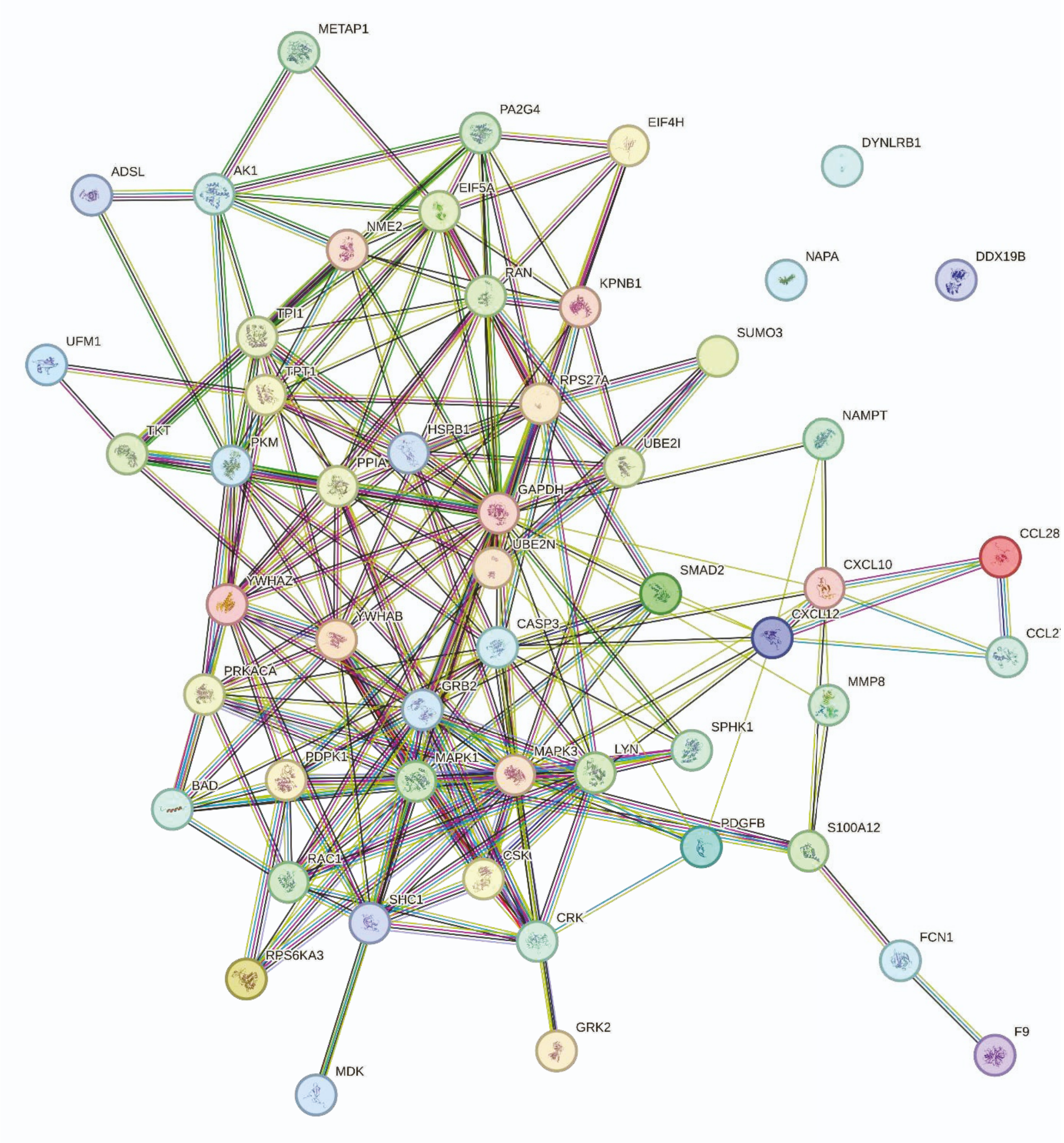

Figure S7. STRING protein-protein network of co-regulated proteins associated with *SNORD12C*. STRING protein–protein interaction network constructed from proteins significantly correlated with *SNORD12C* expression in plasma (n = 1,195 proteins screened; 55 significant associations).

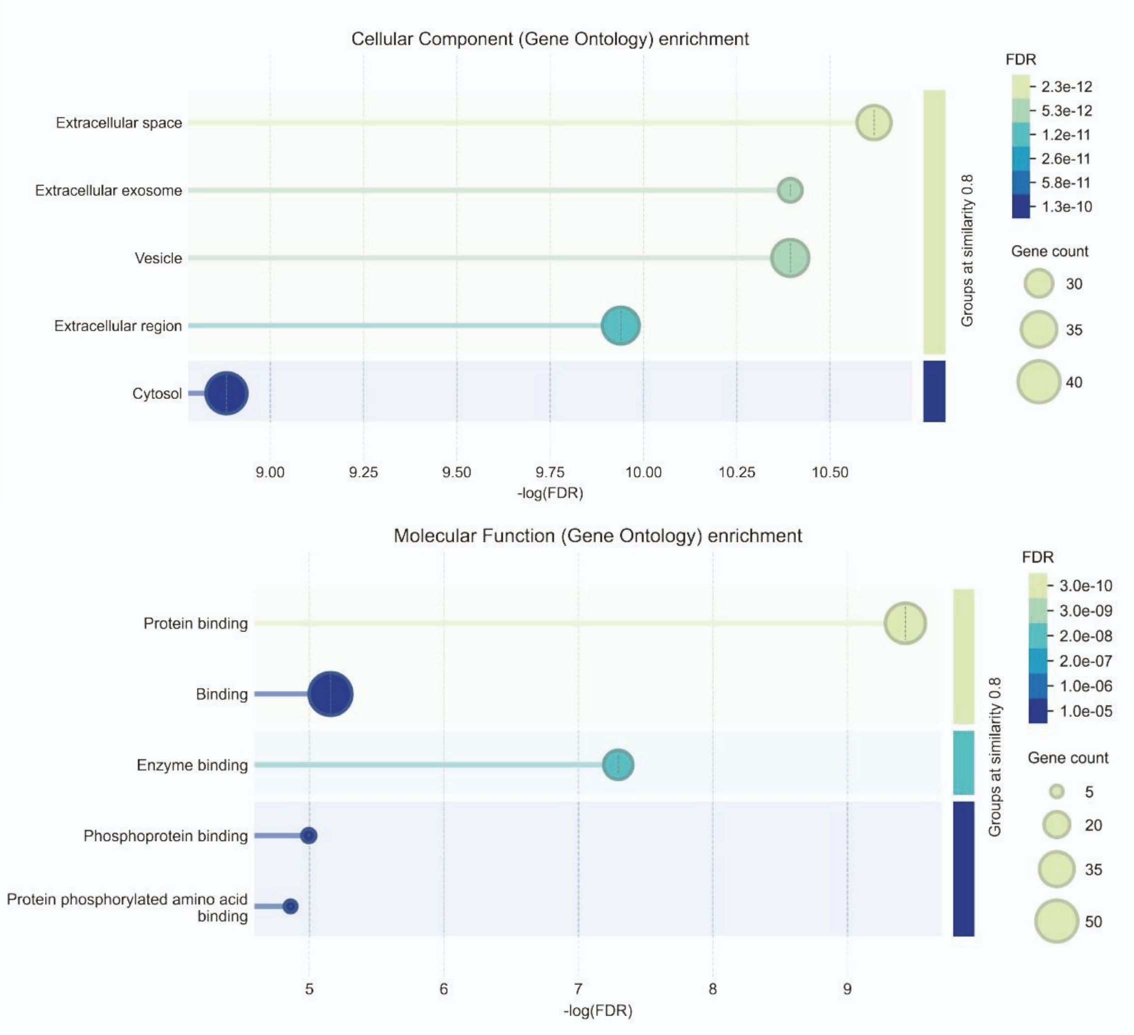

Figure S8. Enriched pathways associated with co-regulated proteins of *SNORD12C*. Correlation analysis between *SNORD12C* expression and 1,195 plasma proteins identified 55 proteins co-regulated with *SNORD12C*. Pathway enrichment analysis of *SNORD12C*-associated proteins revealed significant enrichment for extracellular space/region localization and for binding-related functions, most notably protein binding.

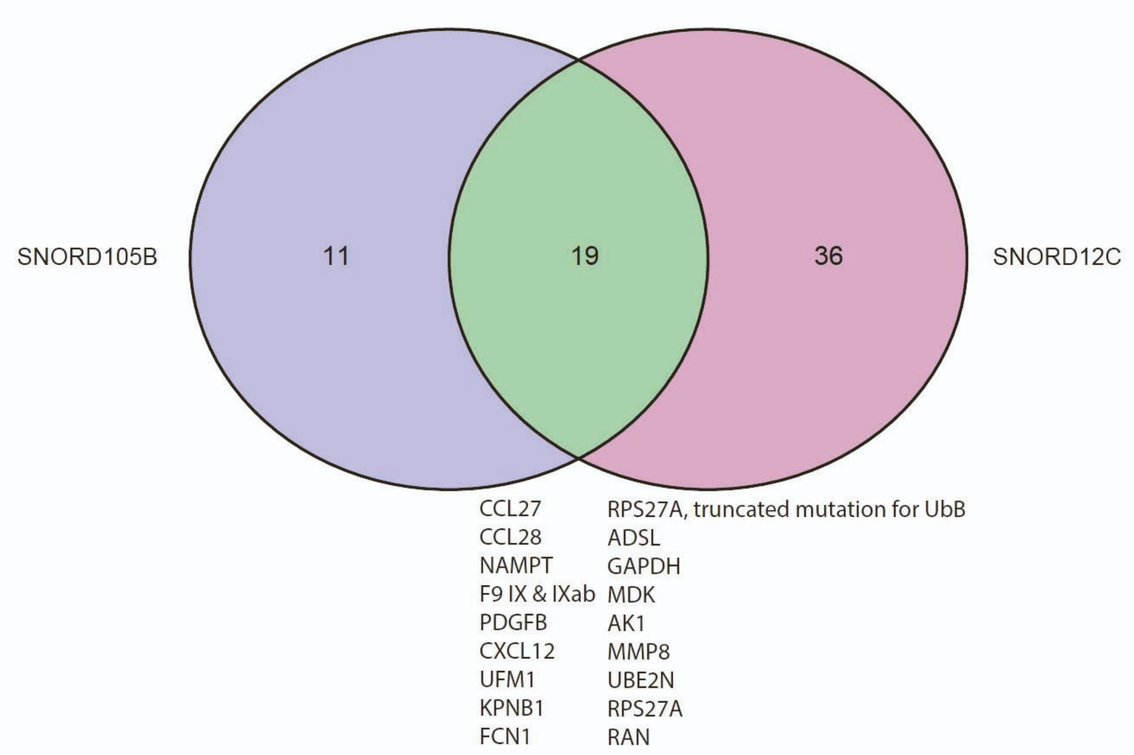

Figure S9. Venn diagram showing the overlap of plasma proteins co-regulated with *SNORD105B* and *SNORD12C*. Correlation analysis between snoRNA expression and 1,195 plasma proteins identified 30 proteins associated with *SNORD105B* and 55 with *SNORD12C*. Nineteen proteins were shared between both snoRNAs: *CCL27*, *CCL28*, *NAMPT*, *F9 IX* and *IXab*, *PDGFB*, *CXCL12*, *UFM1*, *KPNB1*, *FCN1*, *RPS27A truncated mutation for UbB*, *ADSL*, *GAPDH*, *MDK*, *AK1*, *MMP8*, *UBE2N*, and *RPS27A*.

Table S1. Characteristics of the individuals used for the incident CKD study. Data are mean (SD), median (IQR) or n (%)

Patient characteristics, Data are median (IQR) or n (%). *P ≤ 0.05 versus those without CKD			
	**Not CKD (n=122)**	**CKD (n=141)**	**Total (n=263)**
Age (years)	62.8 (57.4 - 68.7)	65.7 (61.9 - 70.1)*	64.5 (59.3 - 69.5)
Female	41.8% (n=51)	44.0% (n=62)	43.0% (n=113)
BMI	28.7 (26.3 - 31.8)	29.8 (27.4 - 33.3)*	29.3 (26.8 - 32.6)
HbA1c (mmol/mol)	48.0 (43.0 - 56.3)	48.6 (44.3 - 56.3)	48.6 (43.2 - 56.3)
HbA1c percentage	6.5% (6.1% - 7.3%)	6.6% (6.2% - 7.3%)	6.6% (6.1% - 7.3%)
Fasting glucose (mmol/L)	8.0 (6.8 - 9.2)	7.8 (7.0 - 9.2)	7.9 (7.0 - 9.2)
Age at diabetes diagnosis (years)	53.9 (49.7 - 58.7)	56.8 (52.2 - 62.0)	55.0 (50.7 - 61.0)
Diabetes duration (years)	8.4 (4.4 - 12.8)	8.0 (4.3 - 12.0)	8.3 (4.3 - 12.8)
Smoking status (% smoking)	18.0 (n=22)	10.6% (n=15)	14.1% (n=37)
HDL (mmol/L)	1.2 (1.0 - 1.5)	1.1 (1.0 - 1.3)	1.2 (1.0 - 1.4)
LDL (mmol/L)	2.4 (2.0 - 3.2)	2.5 (1.9 - 3.1)	2.4 (2.0 - 3.1)
Total Cholesterol (mmol/L)	4.5 (3.9 - 5.2)	4.5 (3.9 - 5.3)	4.5 (3.9 - 5.2)
Triglycerides (mmol/L)	1.5 (1.1 - 2.0)	1.7 (1.3 - 2.3)	1.6 (1.2 - 2.2)
SBP (mmHg)	140.5 (126.0 - 150.0)	145.0 (134.0 - 157.0)*	143.0 (131.5 - 154.0)
DBP (mmHg)	78.0 (72.0 - 84.0)	77.0 (72.0 - 82.0)	77.0 (72.0 - 83.0)
MAP (mmHg)	98.3 (90.4 - 105.3)	99.7 (94.0 - 106.7)	99.3 (92.3 -106.0)
eGFR (mL/min/1.73m2)	88.6 (80.8 - 95.4)	75.2 (68.1 - 86.4)*	83.1 (72.8 - 91.4)
Creatinine blood (µmol/L)	72.0 (64.0 - 82.0)	81.0 (71.0 - 92.0)*	77.0 (67.0 - 88.0)
UACR	0.4 (0.0 - 0.6)	0.7 (0.4 - 1.2)*	0.5 (0.3 - 0.9)
Time until CKD diagnosis (years)	-	6.1 (4.9 - 8.9)	-
Medication use			
Metformine	75.9% (n=107)	67.2% (n=82)	71.9% (n=189)
Sulfonylureas	36.9% (n=52)	27.9% (n=34)	32.7% (n=86)
Insulin	33.3% (n=47)	29.5% (n=36)	31.6% (n=83)
Oral hypoglycemic agents	80.1% (n=113)	73.8% (n=90)	77.2% (n=203)
Other diabetes medication	10.6% (n=15)	3.3% (n=4)*	7.2% (n=19)

Table S2. RNA biotypes incident CKD study. Different biotypes used for the study.

Biotype			
**Name**	**Abbreviation**	**Frequency**	**Percentage**
Micro RNA	microRNA	606	68.8
Small nucleolar RNA	snoRNA	69	7.8
Long non-coding RNA	lncRNA	62	7.0
Miscellaneous RNA	miscRNA	44	5.0
Piwi-interacting RNA	piRNA	35	4.0
Transfer-RNA	tRNA	25	2.8
Circular RNA	circRNA	16	1.8
Small nuclear RNA	snRNA	10	1.1
Small Cajal body-specific RNA	scaRNA	6	0.7
TEC RNA	TEC	4	0.5
Ribozyme	ribozyme	2	0.2
Vault RNA	vault RNA	1	0.1
Ribosomaal RNA	rRNA	1	0.1

Table S3. Result DGE analyse incident CKD. Yellow boxes indicate FDR significant associations.
This table is provided as a separate Excel file due to its size.

Table S4. Result DGE analyse eGFR <60 ml/min. Yellow boxes indicate FDR significant associations.
This table is provided as a separate Excel file due to its size.

Table S5. Result DGE analyse uACR >3 mg/mmol. Yellow boxes indicate FDR significant associations.
This table is provided as a separate Excel file due to its size.

Table S6. Characteristics of the individuals used for the SGLT2 inhibition treatment study. Data are mean (SD), median (IQR) or n (%).
This table is provided as a separate Excel file due to its size.

Table S7. RNA biotypes SGLT2 inhibitor treatment study. Different biotypes used for the study.

Biotype			
**Name**	**Abbreviation**	**Frequency**	**Percentage**
Micro RNA	microRNA	728	65.4
Long non-coding RNA	lncRNA	105	9.4
Miscellaneous RNA	miscRNA	72	6.5
Small nucleolar RNA	snoRNA	71	6.4
Circular RNA	circRNA	39	3.5
Piwi-interacting RNA	piRNA	38	3.4
Transfer-RNA	tRNA	24	2.2
Small nuclear RNA	snRNA	14	1.3
Ribosomaal RNA	rRNA	9	0.8
Small Cajal body-specific RNA	scaRNA	8	0.7
TEC RNA	TEC	4	0.4
Vault RNA	vault RNA	1	0.1
Ribozyme	ribozyme	0	0.0

Table S8. Result DGE analyse SGLT2 inhibitor treatment. Yellow boxes indicate P-value significant associations.
This table is provided as a separate Excel file due to its size.

Table S9. Result sensitivity analysis RED (metformine + dapagliflozin) (n=24). Yellow boxes indicate P-value significant associations.
This table is provided as a separate Excel file due to its size.

Table S10. Result sensetivity analysis RACE_1 (metformine + empagliflozine) (n=20). Yellow boxes indicate P-value significant associations.
This table is provided as a separate Excel file due to its size.

Table S11. Result sensetivity analysis RACE_2 (metformine and linagliptine + empaglilozine). Yellow boxes indicate P-value significant associations.
This table is provided as a separate Excel file due to its size.

Table S12. Co-regulated proteins with *SNORD105B* and *SNORD12C*. Yellow boxes indicate overlap of co-regulated proteins of *SNORD105B* and *SNORD12C*. Sorted by strongest correlation.
This table is provided as a separate Excel file due to its size.

Table S13. GEO accession numbers of public datasets used for small RNA expression in different tissues.

Tissue	Number	GEO ID
Kidney	12	GSE24457
Thyroid	12	GSE79727
Pancreas	5	MTAP-3494
Colon	14	GSE66208
Liver	4	GSE69825
Heart	11	GSE69825,GSE36759
Muscle	12	GSE66334
Brain	17	GSE69825,GSE78812,GSE111623
SC fat	57	PRJNA565427
Whole blood	56	GSE69825,GSE46579
Urine	47	GSE128359
